# The Development of Online Doctor Reviews in China: An Analysis of the Largest Online Doctor Review Website in China

**DOI:** 10.2196/jmir.4365

**Published:** 2015-06-01

**Authors:** Haijing Hao

**Affiliations:** ^1^University of Massachusetts BostonDepartment of Management Science and Information SystemsBoston, MAUnited States

**Keywords:** online doctor reviews, China health system, quantitative review, qualitative review, patient empowerment, physician quality

## Abstract

**Background:**

Since the time of Web 2.0, more and more consumers have used online doctor reviews to rate their doctors or to look for a doctor. This phenomenon has received health care researchers’ attention worldwide, and many studies have been conducted on online doctor reviews in the United States and Europe. But no study has yet been done in China. Also, in China, without a mature primary care physician recommendation system, more and more Chinese consumers seek online doctor reviews to look for a good doctor for their health care concerns.

**Objective:**

This study sought to examine the online doctor review practice in China, including addressing the following questions: (1) How many doctors and specialty areas are available for online review? (2) How many online reviews are there on those doctors? (3) What specialty area doctors are more likely to be reviewed or receive more reviews? (4) Are those reviews positive or negative?

**Methods:**

This study explores an empirical dataset from Good Doctor website, haodf.com—the earliest and largest online doctor review and online health care community website in China—from 2006 to 2014, to examine the stated research questions by using descriptive statistics, binary logistic regression, and multivariate linear regression.

**Results:**

The dataset from the Good Doctor website contained 314,624 doctors across China and among them, 112,873 doctors received 731,543 quantitative reviews and 772,979 qualitative reviews as of April 11, 2014. On average, 37% of the doctors had been reviewed on the Good Doctor website. Gynecology-obstetrics-pediatrics doctors were most likely to be reviewed, with an odds ratio (OR) of 1.497 (95% CI 1.461-1.535), and internal medicine doctors were less likely to be reviewed, with an OR of 0.94 (95% CI 0.921-0.960), relative to the combined small specialty areas. Both traditional Chinese medicine doctors and surgeons were more likely to be reviewed than the combined small specialty areas, with an OR of 1.483 (95% CI 1.442-1.525) and an OR of 1.366 (95% CI 1.337-1.395), respectively. Quantitatively, traditional Chinese medicine doctors (*P*<.001) and gynecology-obstetrics-pediatrics doctors (*P*<.001) received more reviews than the combined small specialty areas. But internal medicine doctors received fewer reviews than the combined small specialty areas (*P*<.001). Also, the majority of quantitative reviews were positive—about 88% were positive for the doctors' treatment effect measure and 91% were positive for the bedside manner measure. This was the case for the four major specialty areas, which had the most number of doctors—internal medicine, gynecology-obstetrics-pediatrics, surgery, and traditional Chinese medicine.

**Conclusions:**

Like consumers in the United States and Europe, Chinese consumers have started to use online doctor reviews. Similar to previous research on other countries’ online doctor reviews, the online reviews in China covered almost every medical specialty, and most of the reviews were positive even though all of the reviewing procedures and the final available information were anonymous. The average number of reviews per rated doctor received in this dataset was 6, which was higher than that for doctors in the United States or Germany, probably because this dataset covered a longer time period than did the US or German dataset. But this number is still very small compared to any doctor’s real patient population, and it cannot represent the reality of that population. Also, since all the data used for analysis were from one single website, the data might be biased and might not be a representative national sample of China.

## Introduction

### Overview

Online doctor reviews have been happening across the world since the Internet Web 2.0 came into use in the early 2000s, and they have attracted health care researchers’ attention about how these reviews have been used in different countries [1]. Based on a study conducted in 2012, about 17% of physicians had been rated on the Internet in the United States [2]. In the United Kingdom, about 61% of family physicians who are posted on the National Health Service website have been rated [3]. In Germany, 37% of German physicians were rated on the jameda website in 2012 [4]. Also, the difference between traditional patient reviews of their doctors and the online doctor reviews has been discussed [4], as well as what type of information the online doctor reviews could provide [5]. Further, some research also examined the online reviews in different medical specialty areas, such as dentistry [6] and orthopedics [7]. Some research also raised concerns about online doctor reviews, which may be subject to manipulation or could damage physicians’ reputations [8-12]. At the same time, studies about how health care consumers used the online doctor reviews have been conducted. A cross-sectional survey in Germany showed that about 32% of respondents heard of physician-rating websites, and about 25% had already used a website to search for a physician [13]. A survey conducted via the most popular online social network in the Netherlands found that about one-third of the Dutch population searched for ratings of health care providers [14]. A representative sample of citizens who were at least 15 years old from seven European countries were surveyed. The results showed that among the people who use the Internet for health care-related purposes, on average, more than 40% of people considered the information of these eHealth services to be important when choosing a new doctor [15]. A 2012 survey in the United States showed that 17% of Internet users have consulted physician-rating sites, and 4% of people posted a review online of a doctor [16]. A 2012 study comprised of a nationally representative sample of US citizens found that 59% of the survey respondents said that online doctor ratings are “somewhat important” for them and 19% said they are “very important” for them when they search for a physician [17]. A study also examined what factors may affect consumers’ decisions to adopt online doctor reviews [18].

We can see that various studies regarding online doctor reviews, either based on secondary data on how many doctors have been reviewed online or based on the first-hand survey data on how patients look at those online doctor reviews, have been emerging in the United States and Europe, but there has been no study about whether Chinese consumers use the Internet to review their health care providers. Considering the fact that China has the largest population of Internet users in the world [19], and China is already known for having more than 1 million online doctor reviews by international media [20], this study wants to examine further the current status of online doctor reviews in China.

Without a mature primary care system in China, most Chinese consumers now largely have to self-refer to any health care provider they can afford or who they believe is good based on little to no information [21]. Internet technology brings a new option for Chinese consumers and, particularly, Web 2.0 technology brings the interactive form of information sharing online. The first online local service review website in China was established in 2003—General Public Review Web. At the end of 2014, there were more than 60 million public reviews on General Public Review Web and most of those reviews were about local restaurants or movie theaters [22]. Based on the searches conducted on Google and Baidu, the number one search engine in China [23], and personal meetings with medical professionals in Beijing, the author found a few online doctor review websites in China. Chinese Traditional Medicine Review Web (zydp.org) [24], established in December 2013, focuses completely on reviewing traditional Chinese medicine doctors. Schedule Appointment website (guahao.com) [25], established in 2010, claims that its goal is to help patients to schedule doctor appointments online. At the same time, it also provides a feature to review doctors online. Hao Dai Fu, or the Good Doctor website (haodf.com)—hao means “good” and dai fu means “doctor” in Chinese [26]—is the earliest online doctor review website in China, established in 2006. Its purpose is to provide an online doctor reviewing system for Chinese consumers to review their doctors, and it also helps consumers to select a good doctor for their health care concerns based on online reviews. Also, the Good Doctor website already had more than 1 million reviews by the summer of 2014 [20].

Thus, the purpose of this study is to examine the following research questions about the current status of online doctor reviews in China based on the Good Doctor website: (1) How many doctors and how many specialty areas are available to be rated online on the Good Doctor website? (2) Which medical specialties are most likely to be rated? (3) Which medical specialties receive more reviews? (4) How are the quantitative rating scores distributed? and (5) What are the developing trends of online doctor reviews on the Good Doctor website?

### Background

According to meetings with Mr Hang Wang, the founder of the Good Doctor website, the original purpose in establishing this website was to help Chinese consumers to find good and appropriate specialists for their health care problems based on online reviews, after he personally experienced difficulty in finding a good specialist doctor in Beijing. In 2006, the Good Doctor website was launched in Beijing, China, and for the first time an online doctor review system became available for Chinese consumers.

Since its establishment, the Good Doctor company staff has been manually collecting information on Chinese doctors and their hospitals by various means—in China, a majority of doctors work at public hospitals where they are employees and have a responsibility for both inpatient service and outpatient service. The staff collected information by visiting hospital campuses in person, making phone calls to hospitals, or searching hospital websites, for those hospitals that had them. The staff then posted the collected information about the doctors on the Good Doctor website for consumers to browse and review for free. They knew that they would not have a national database to rely on and, as a start-up, they had limited human resources. Therefore, their strategy was to start with the largest and most famous hospitals in Beijing and Shanghai, then gradually cover the remaining parts of China, since the most reputable Chinese hospitals are concentrated mostly in Beijing or Shanghai. According to Fudan University’s hospital ranking system, which was based on a peer-review system on hospitals’ medical practice, quality of care, and research [27], 26 out of the 100 best hospitals are in Beijing, and 19 are in Shanghai. The posted information includes each doctor’s name, short biography, specialty area, technical title, and hospitals where they work. Chinese doctors have a technical title system and the title is assigned through an evaluation process. The rank is nationally unified as four levels—from junior to senior levels—from Resident Physician, Attending Physician, Associate Physician, to Chief Physician. On average, every 5 years a doctor can move up one level in this title track. Thus, a title primarily indicates a doctor’s work experience and technical skills, which also determines the consultation fee of a doctor. The Good Doctor website also posts the hospital information where doctors work, such as the hospital name, address, and grade of the hospital. China’s hospital grades are evaluated by a government agency—the National Health Department at the provincial level—and the evaluation standards are based on the hospital facilities, the number of beds, technical equipment, quality of care, the doctors’ skills, etc [28].

Once a doctor’s information is posted on the Good Doctor website, patients can anonymously review those doctors online based on their inpatient or outpatient experiences with the doctor. There are two types of reviews on the Good Doctor website: one is a quantitative review with two measures, *treatment effect* and *bedside manner*, which have to be done together, and the other is a narrative open-ended textual review, which can be done separately from the quantitative reviews. Both of the quantitative measures use a 5-level rating scale, from Very Unsatisfied (1), Unsatisfied (2), OK (3), Satisfied (4), to Very Satisfied (5). Over a few years of development, besides the doctor rankings by specialty area based on patients' reviews, the Good Doctor website has also developed other features. These features include the following: a doctor’s personal webpage on the Good Doctor website where the doctor can post medical articles or health care advice, a doctor’s personal forum where patients can post questions or initiate discussions with doctors that they choose, daily updates of a doctor’s outpatient schedule, appointment scheduling online, telephone consultation, and membership in private patient clubs, etc.

## Methods

### Data

Based on the application programming interface (API) provided by the Good Doctor website, this study collected data on 314,624 doctors and their associated 3091 hospitals from the website as of April 11, 2014. After data cleaning by removing the records with missing values or abnormal values, there were 731,316 quantitative reviews, including both *treatment effect* and *bedside manner*, and 772,979 narrative textual reviews on 117,624 doctors across China from almost every specialty area. A total of 731,264 records had both quantitative and qualitative reviews. This study focuses on the two 5-scale quantitative reviews only.

Based on the dataset from the Good Doctor website, there are nine major specialty areas and one specialty area called “others,” which groups all the uncommon, small specialty areas not listed separately on the Good Doctor website. Table 1 shows that traditional Chinese medicine, gynecology-obstetrics-pediatrics, internal medicine, and surgery are the four top specialty areas which have the most number of doctors and had the most number of reviews, excluding "others" because it is not a single specialty area. Also, those four major specialty areas consist of 8.7%, 12.1%, 21%, and 18.3% of the total doctor population on the Good Doctor website, respectively, which is similar to the national composition of the doctors by percentage of those four specialty areas—16%, 15%, 20.7%, and 12.9%, respectively [29]. Therefore, this study selected those four specialty areas as the major focus for analysis.

**Table 1:** Specialty areas, number of doctors, and number of reviews from the Good Doctor website.

Specialty areas	Total doctors, n	Doctors receiving reviews, n (%)	Total reviews, n	Average reviews per rated doctor
Cancer	2781	1317 (47.36)	7018	5.3
Traditional Chinese medicine^a^	27,299	12,011 (44.00)	85,649	7.1
Gynecology-obstetrics-pediatrics^a^	38,099	16,506 (43.32)	122,073	7.4
Infectious disease	1122	483 (43.05)	2869	5.9
Internal medicine^a^	66,162	22,345 (33.77)	96,892	4.3
Orthopedics	1008	495 (49.11)	3592	7.3
Others	112,483	36,038 (32.04)	226,823	6.3
Psychiatry	2848	1050 (36.87)	5800	5.5
Oral health	5106	2671 (52.31)	15,690	5.9
Surgery^a^	57,716	24,708 (42.81)	164,910	6.7
Total	314,624	117,624 (37.39)	731,316	6.2

^a^Specialty area that is among the four top specialty areas, which have the most number of doctors.

Table 2 shows the number of reviews for each specialty area in each year. This study ignored 2006 and 2014 data because those two years were not complete calendar years in the dataset. We can see that the number of reviews per year has been increasing over time for all specialty areas, except for 2010 and 2013, both of which had a little dip for all but one specialty area, oral health.

**Table 2:** Number of reviews for each specialty per year.

Year	Cancer	TCM^a^	G-OB-P^b^	ID^c^	IM^d^	OP^e^	Others	Psychiatry	Oral health	Surgery
2006	0	20	115	1	142	0	155	5	13	108
2007	387	3351	6745	143	6335	143	11,055	261	642	7912
2008	691	10,114	16,008	415	12,508	334	26,006	659	1392	17,177
2009	1000	12,930	18,619	454	13,336	476	33,003	809	1849	21,623
2010	790	10,349	13,788	309	10,081	361	25,393	655	1900	18,830
2011	1181	15,186	20,185	520	14,755	671	38,650	954	2571	27,702
2012	1365	17,676	22,491	531	18,568	846	43,997	1212	3207	33,057
2013	1292	13,561	19,033	388	16,536	605	38,705	968	3222	30,134
2014	312	2462	5089	108	4631	156	9859	277	894	8367
Total	7018	85,649	122,073	2869	96,892	3592	226,823	5800	15,690	164,910

^a^Traditional Chinese medicine (TCM)

^b^Gynecology-obstetrics-pedicatrics (G-OB-P)

^c^Infectious disease (ID)

^d^Internal medicine (IM)

^e^Orthopedics (OP)

### Statistical Analysis

In order to further examine the research question of which types of doctors are more likely to be rated, a binary logistic regression model was constructed as follows:

Logit (Rated_i_) = Specialty Area_i_ + Physician Title_i_ + Hospital Level_i_ + Beijing_i_ + Shanghai_i_ (1)

Rated_i_ equals 1 if doctor i has been rated, otherwise it is 0. Specialty Area_i_ is a categorical variable which differentiates the four major specialty areas from the rest of the combined specialty areas, combined specialties. The combined specialties combined the other five specialty areas listed by the Good Doctor website—infectious disease, orthopedics, psychiatry, oral health, and cancer—with the “others” for concision. Physician Title_i_ is a categorical variable, too, which indicates doctor i’s technical title from one of the four levels that was discussed earlier. There are three levels of hospital grades—Level 3 is the highest with more beds, better equipment, more highly skilled doctors, etc. This model also controls for Beijing and Shanghai because higher-ranking hospitals are more concentrated in these two cities than in other areas in China.

The following model examines which specialty area doctors would receive more ratings by using a multivariate linear regression model:

Rating_count_i_ = Specialty Area_i_ + Physician Title_i_ + Hospital Level_i_ + Beijing_i_ + Shanghai_i_ + error_i_ (2)

The dependent variable, Rating_count_i_ , is the number of ratings doctor i received. The independent variables are similar to those in model (1) for doctor i’s specialty area, technical title, hospital level, and whether the hospital is in Beijing, Shanghai, or another area.

## Results

Regarding the first research question of how many doctors and how many specialty areas are available for review, from Table 1 we can see that there are 314,624 doctors from nine major specialty areas and many small specialty areas combined that are available for online review on the Good Doctor website. Among them, 117,624 doctors have been reviewed since 2006, which is 37.39% of the total doctors available for review. Among those nine major specialty areas, internal medicine has the lowest review percentage at 33.77%, and oral health has the highest review percentage at 52.31%. But since the total number of doctors in oral health is small—only 5106—this study mainly focused on the four major specialty areas, which include the most numbers of doctors: traditional Chinese medicine, gynecology-obstetrics-pediatrics, internal medicine, and surgery. Except for internal medicine, which has a review rate of 33.77%, the other three specialty areas all have a review rate of about 43.32% to 44.00%.

Table 3 shows the binary logistic regression results. We can see that doctors from traditional Chinese medicine, gynecology-obstetrics-pediatrics, and surgery were all about 1.5 times more likely to be reviewed compared to doctors from combined specialties, which is the reference group of the model. Doctors from internal medicine were less likely to be reviewed compared to the doctors from combined specialties. Also, chief physicians were about 4.6 times more likely to be reviewed, associate physicians were about 2.5 times more likely to be reviewed, and attending physicians were 1.6 times more likely to be reviewed than resident physicians. Doctors from Level 3 hospitals were 2 times more likely to be reviewed than doctors from Level 1 hospitals, and doctors from Level 2 hospitals were 1.5 times more likely to be reviewed than doctors from Level 1 hospitals. Doctors in Beijing and Shanghai were 1.5 times and 2 times more likely, respectively, to be reviewed than doctors from other areas of China. All of the estimated odds ratios are statistically significant at a 95% Wald confidence level.

**Table 3:** Results from the binary logistic regression (n=314,624).

Effect (independent variable)^a^	Odds ratio point estimate^b,c,d^	95% Wald CI
Traditional Chinese medicine	1.483	1.442-1.525
Gynecology-obstetrics-pediatrics	1.497	1.461-1.535
Internal medicine	0.940	0.921-0.960
Surgery	1.366	1.337-1.395
Chief physician	4.648	4.525-4.774
Associate physician	2.592	2.526-2.661
Attending physician	1.624	1.576-1.673
Level 3 hospital	2.047	1.995-2.100
Level 2 hospital	1.590	1.548-1.633
Beijing	1.532	1.486-1.579
Shanghai	2.102	2.035-2.172

^a^ *Combined specialties* is the reference group for specialty areas, *resident physician* is the reference group for technical title, *Level 1 hospital* is the reference group for hospital grade, and *other areas* is the reference group for Beijing and Shanghai.

^b^Pseudo R^2^ = .115.

^c^The dependent variable is *reviewed or not*.

^d^5% significance level for all values.

Table 4 exhibits the linear regression results for which types of doctor would receive more reviews quantitatively. We can see that traditional Chinese medicine, gynecology-obstetrics-pediatrics, and surgeon had positive associations with the number of reviews a doctor received, but internal medicine was negatively associated with the number of reviews a doctor received. A chief physician, on average, can have 6 more reviews than a resident physician, which was the largest impact in this model. There are also positive impacts of being an associate physician or an attending physician on the number of reviews a doctor would receive, but the quantitative scale is smaller than that of a chief physician when comparing all of these to a resident physician. Also, being a doctor in Beijing or Shanghai is associated with 3 or 5 more reviews, respectively, than being a doctor in other areas of China. Being a doctor in a Level 3 hospital was associated with more reviews compared to being a doctor in a Level 1 hospital. But interestingly, a doctor working in a Level 2 hospital may receive fewer reviews compared to a doctor working in a Level 1 hospital. All of the estimates, except for surgery, are statistically significant at a 5% level.

**Table 4:** Results for linear regression for doctors in different areas receiving reviews (n=117,624).

Independent variable^a^	Parameter coefficient estimate^b,c,d^	Standard error	*t*_11_	*P*
Intercept	2.17	0.24	9.14	<.001
Traditional Chinese medicine	0.62	0.17	3.58	<.001
Gynecology-obstetrics-pediatrics	0.97	0.15	6.34	<.001
Internal medicine	-2.44	0.14	-17.69	<.001
Surgery	0.26	0.13	1.95	.052
Chief physician	6.43	0.19	33.59	<.001
Associate physician	2.54	0.19	13.29	<.001
Attending physician	0.93	0.22	4.22	<.001
Level 3 hospital	0.53	0.17	3.10	.002
Level 2 hospital	-1.29	0.18	-7.15	<.001
Beijing	3.23	0.18	18.01	<.001
Shanghai	5.37	0.18	29.28	<.001

^a^ *Combined specialties* is the reference group for specialty areas, *resident physician* is the reference group for technical title, *Level 1 hospital* is the reference group for hospital grade, and *other cities* is the reference group for Beijing and Shanghai.

^b^Adjusted R^2^=.0353.

^c^The dependent variable is the *number of reviews*.

^d^5% significance level for all values.

**Figure 1:**
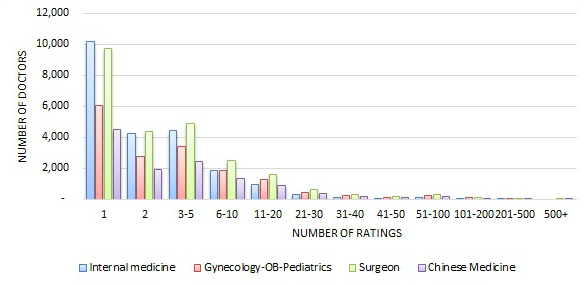
Distribution of the number of ratings across four major specialty areas (absolute number).

**Figure 2:**
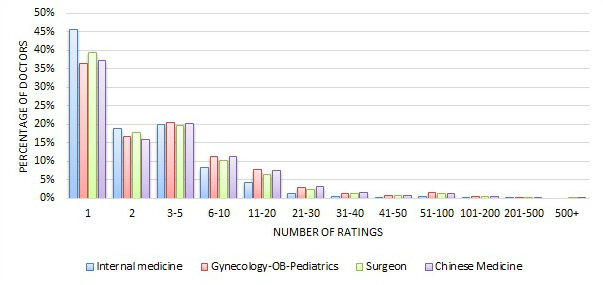
Distribution of the number of ratings across four major specialty areas (relative percentage).

Figures 1 and 2 show the distribution of the number quantitative ratings a doctor received by the four major specialty areas, in absolute numbers and relative percentages. The distributions for the four specialty areas were quite similar—about 37% to 45% of doctors received 1 review, about 16% to 19% of doctors received 2 reviews, and about 19% to 21% of doctors received 3 to 5 reviews. In a few extreme cases, some doctors had received more than 500 reviews. Therefore, the number of quantitative reviews a doctor received was quite spread out.

**Figure 3:**
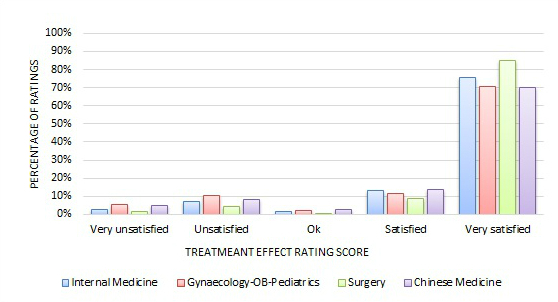
Distribution of treatment effect ratings across four major specialty areas.

**Figure 4:**
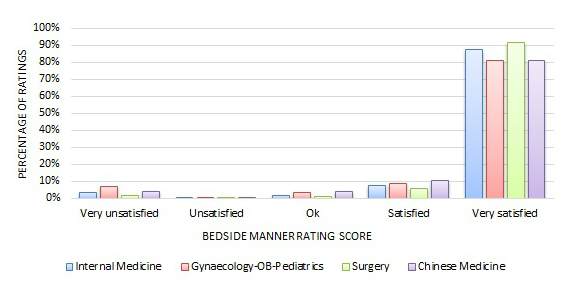
Distribution of bedside manner ratings across four major specialty areas.

Figures 3 and 4 show the quantitative rating score distribution among the four major specialty areas. We can see, regardless of the specialty area, that most quantitative ratings were positive—82% to 95% of reviews had responses of either Satisfied or Very Satisfied on either the *treatment effect* or *bedside manner* measure.

**Figure 5:**
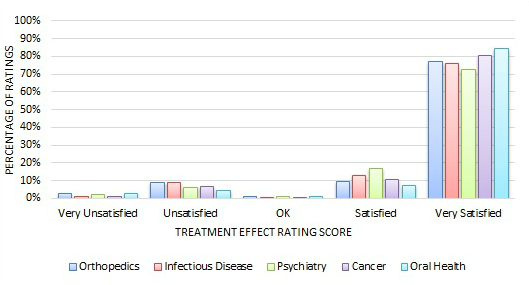
Distribution of treatment effect ratings across small specialty areas.

**Figure 6:**
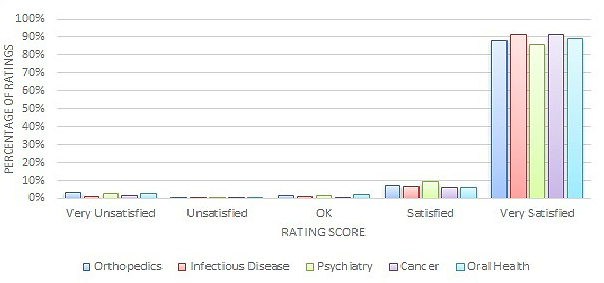
Distribution of bedside manner ratings across small specialty areas.

Figures 5 and 6 display the quantitative rating score distribution for the other small five specialty areas for *treatment effect* and *bedside manner*, respectively. Again, the quantitative reviews highly concentrate at the positive end of the rating scores.

**Figure 7:**
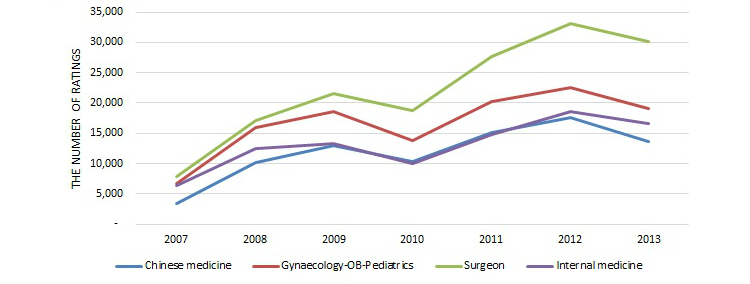
Number of ratings over time among four major specialty areas.

**Figure 8:**
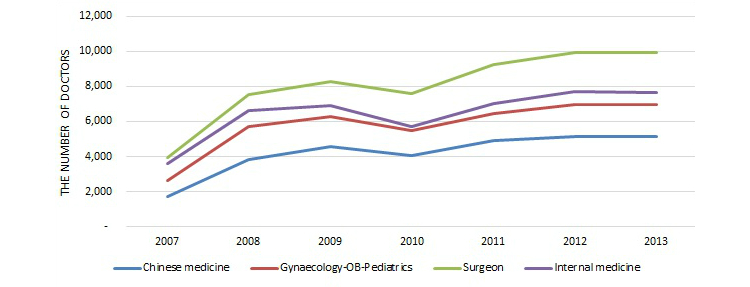
Number of doctors rated over time among four major specialty areas.

**Figure 9:**
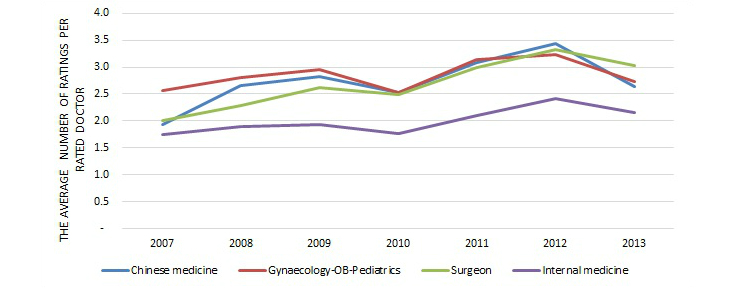
Average number of ratings per rated doctor over time among four major specialty areas.

Based on Table 2 and Figure 7, which shows the number of ratings over time, we can see that the number of reviews on the Good Doctor website has been growing for all specialty areas over the years, except for a little dip in years 2010 and 2013. The number of rated doctors had been growing, then stayed relatively stable in 2012 and 2013, with a similar little dip in 2010, as Figure 8 shows. The average number of ratings per doctor over time was relatively stable, within the range of 1.8 to 3.4, as seen in Figure 9. Since this study is based on a secondary dataset, further investigation is needed to determine the reason for those specific dips.

## Discussion

### Principal Findings

First, we should realize that this study examines a dataset from a single website in China. Compared to developed countries, such as the United States, the United Kingdom, or Germany, the disadvantage of a dataset from a developing country like China is a lack of an official database which could be accessed to obtain the number of doctors in each specialty area at either the hospital level, the provincial level, or the national level. Thus, it is difficult to stratify this dataset to make a nationally representative sample. But the current dataset is an empirical dataset from the earliest and the largest available online doctor review website in China, and the four major specialty areas’ percentage compositions are close to the national level aggregated data. Therefore, this empirical dataset will assist us to understand the current status of online doctor reviews in China.

The average review rate of all doctors over about 8 years on the Good Doctor website was 37.4%, which is close to the national review rate in Germany in 2012 of 37% [4], higher than the review rate between 2005 and 2010 in the United States of 17% [2], and lower than the review rate of family practice physicians in the United Kingdom of 61% between 2009 and 2012 [3]. But it should be noted throughout this study that it was difficult to compare across those countries because the datasets from different countries were collected by different means, and the sample sizes or time periods of those datasets were different.

On the Good Doctor website, 43% of gynecology-obstetrics-pediatrics doctors had been reviewed, which was higher than the review rate of obstetricians in the United States of 33% [2], but lower than that of gynecologists in Germany of 56.9% [4]. We should note that each country’s categorization for specialty area is a little different. The Good Doctor website groups gynecology, obstetrics (OB), and pediatrics as one specialty area. Germany studied gynecology and the United States focused on obstetrics. Thus, that might be a reason why the review rate in China is higher because it covers more specialty areas compared to the United States or Germany. If we look further, statistically, at which specialty areas were more likely to be reviewed by using the logistic regression model, we can see that gynecology-obstetrics-pediatrics was the specialty area most likely to be reviewed in China. It was 1.5 times more likely to be reviewed than all of the other small medical specialties combined, which is similar to a study in the United States that showed the OB specialty was more likely to be rated, compared to other specialty areas [2]. This may indicate, as that study suggested, that obstetrics patients—similarly for gynecology or pediatrics patients in this study—are a mostly young and female population who are more likely to log on and use the Internet [2]. A study in Germany also showed that more women than men had used online doctor review websites [30]. Another possible reason that Chinese gynecology-obstetrics-pediatrics doctors received more reviews might be that children are always the focus of a family and the extended family in Chinese culture, thus consumers paid more attention to the quality of care by these doctors. Previous research also showed that the length of the relationship between a doctor and their patients plays a role in online doctor reviews, and patients who have had a longer relationship with their doctors would be more likely to review their doctors [31]. Not surprisingly, most patients would have a relatively stable and longer relationship with their gynecology-obstetrics-pediatrics doctors than with doctors of other medical specialties, hence they would be more likely to review these doctors. The Chinese dataset used in this study also showed that other major specialty areas—surgery and traditional Chinese medicine—received a similar, higher review rate as that of gynecology-obstetrics-pediatrics, which is a little different from the US and the German data. Surely, traditional Chinese medicine is a mainstream medical specialty only in China. Also, traditional Chinese medicine doctors mainly practice in herbal medicine, which usually has a longer treatment time and is not used for acute diseases. Thus, higher review rates might be due to the length of the patient-doctor relationship, too. Surgeons received more reviews probably because they usually have a longer and more interactive relationship with their patients. But the real reasons why doctors from those specialty areas were more likely to be reviewed need further investigation with richer data.

Doctors from the Beijing or Shanghai areas were more likely to be reviewed and were also likely to receive more reviews than doctors from other areas in China. This might be because, first, Beijing and Shanghai have more famous hospitals than other parts of China [27] which attract not only local patients, but patients nationwide. If a patient is nonlocal, the patient probably has a more serious or uncommon disease requiring them to travel to Beijing or Shanghai. If so, these types of patients are more serious about their doctors and probably more likely to review their doctors. Second, local residents of the metropolitan areas of Beijing or Shanghai have the highest Internet accessibility in China—75% and 70%, respectively—compared to other areas where Internet accessibility is lower than 66% [32]. Therefore, local patients have more Internet access and may be more likely to review their doctors. Thus, the doctors from Beijing or Shanghai would be more likely to be reviewed.

Doctors from Level 3 hospitals were more likely to be reviewed, and were likely to receive more reviews than doctors from the Level 1 hospitals. Level 3 hospitals usually have more beds, better equipment, more highly skilled doctors, and deal with more challenging diseases. Again, this might suggest that patients with more serious health care problems and probably a longer interaction time are more likely to review their doctors. Interestingly, doctors from Level 2 hospitals were more likely to be reviewed than doctors from Level 1 hospitals, but received fewer reviews quantitatively compared to Level 1 hospitals. Specific reasons for this phenomenon needs research and data. But one thing that we should realize is that the Good Doctor website intentionally started their data collection from Level 3 hospitals or famous hospitals from large metropolitan areas in order to help consumers find good specialists. This strategy may have resulted in sample selection problems because on the Good Doctor website, 54% of the doctors were from Level 3 hospitals and 38% of the doctors were from Level 2 hospitals. Compared to the national data, where 49% of doctors work in Level 3 hospitals and 51% of doctors work in Level 2 hospitals (there are no Level 1 hospitals in the national aggregated data), the aggregated compositions are different. In other words, the national statistics indicate that the number of doctors in Level 3 and Level 2 hospitals are close in quantity but the Good Doctor website collected more doctors’ information from Level 3 hospitals to post online for patients to review.

Although, in total, there were more than 700,000 reviews in either the quantitative or the qualitative review sets, the average number of reviews per rated doctor was about 6.2, compared to that of the United States, which is 3 [2], and Germany, which is 2.37 [4]. We should point out that the dataset from the Good Doctor website covers a longer time period than the datasets from either the United States or Germany. If we look at the distribution of the number of reviews that doctors received for the four major specialty areas only (Figure 2), we can see that about 37% to 45% of doctors who received reviews received only 1 review, which is a little lower than that of Germany where 49.7% of physicians received a single review [4]. That also means that among the doctors who received reviews, a higher percentage of Chinese doctors compared to German doctors received more than 1 review. About 74% to 84% of Chinese doctors received 1 to 5 reviews, which is also a lower rate than that of Germany, where 93.4% of doctors received 1 to 5 reviews. This is also consistent with the fact that German doctors received a lower average number of reviews per doctor than Chinese doctors.

There might be a couple of possible reasons why Chinese consumers would like to review their doctors online. First, without a mature primary care system, Chinese consumers rely more on online doctor reviews to search for a doctor than their Western counterparts. Second, as a developing country, China usually has no formal pen-and-paper-based postvisit surveys to let patients review their health care providers. Some hospitals or clinics may provide a pen-and-paper-based “comment book” in the hospital lobby for patients to leave comments. But this is very informal and most patients ignore the comment book because most hospital lobbies are busy and crowded. Therefore, online reviewing may be the only way, or may be the first time, a Chinese patient can feel free to comment on their doctors with a structured measure. Also, the Good Doctor website was the only online doctor review website in China for a number of years, which may have allowed the Good Doctor website to accumulate more data. Again, further evidence and research are needed to answer the questions of why Chinese patients review their doctors online and how accurate the reviews are.

There has been no study about how Chinese consumers use or look at online doctor reviews or online health care information, and what factors may affect Chinese patients to participate in online doctor reviews. Some research has shown that 59% of American adults used the Internet for health care information and 16% of American adult Internet users have consulted online doctor reviews [33]. A cross-sectional survey conducted in a town in the United Kingdom suggested that the relationship between doctors and their patients may play a role in the patients’ intention to use online doctor review websites [34]. We should expect that more and more studies, either qualitative or quantitative, will investigate what Chinese consumers think about online doctor reviews and how they use them.

Many studies have found that most online doctor reviews are very positive [7,35-38]. Similarly, the majority of the online doctor reviews on the Good Doctor website in China were very positive, too. As Figures 3-6 exhibit, on the Good Doctor website across the four major medical specialties, 88% of the treatment effect evaluations and 91% of the bedside manner evaluations were positive. As well, 75% and 86% of ratings were of the highest level for treatment effect and for bedside manner, respectively. Similar distributions were seen for the five small medical specialties. These were all higher than those of the United States [2] or Germany [4], where 50% and 80% of evaluations, respectively, were in the two best rating categories. Different specialty areas may have variations, but these variations were small on the Good Doctor website. A qualitative study based on a randomly selected sample from online doctor reviewing websites in the United States showed that the majority of online doctor reviews were positive, and in addition to the direct interaction between doctors and patients, staff, access, and convenience all affected patients’ reviews of their doctors [37]. Another study also showed that the UK National Health Service Choice website allowed patients to evaluate their family physicians online and the ratings for all the questions were also quite positive [3]. Thus, Chinese consumers are not different in the positively dominated online reviews of their doctors compared to their Western counterparts, but do seem to give a higher number of, and more positive, evaluations of their doctors. An experimental study in Germany suggested that more reviews may lead to more positive perspectives of a doctor [39]. One research study conducted in a metropolitan area in the United States also showed that a physician’s bedside manner and professional knowledge would significantly lead to a higher rating [7]. Reasons why Chinese patients give more positive reviews could be because of cultural differences or the website review procedures. On the Good Doctor website, although the online evaluation is anonymous to the public, the reviewers are requested to leave a phone number so the webmaster can confirm the truthfulness of the review, if needed. This might lead some conservative people to be cautious, thus they may not leave negative reviews. It’s possible that even the name of the website, Good Doctor, may indicate some signal to the consumers as to the nature of the review they should leave. Again, further studies are needed to investigate why the majority of Chinese online doctor reviewers tend to give very positive online doctor reviews.

### Conclusions

In summary, many Chinese consumers have reviewed their doctors online as their Western counterparts have done. By April 11, 2014, 314,624 doctors from almost every medical specialty in China were listed on the Good Doctor website for Chinese consumers to review. There were 731,316 records of quantitative review, including both treatment effects and bedside manner, and 772,979 records of narrative textual review on 117,624 doctors from nine major specialty areas and many small unlisted specialty areas. The first contribution of this study is that it is the first, or one of the first, studies to examine the current status of online doctor reviews in China. Second, empirically, this study shows that like other countries, online doctor reviews in China covered almost all major medical specialties. Gynecology-obstetrics-pediatrics, surgery, and traditional Chinese medicine were more likely to be reviewed than the combined uncommon specialty areas, and gynecology-obstetrics-pediatrics and traditional Chinese medicine received more reviews than the combined specialty areas. But another major specialty area, internal medicine, was less likely to be reviewed than the combined specialty areas. All of the model estimates, except for surgery for the quantitative reviews, were statistically significant at the 5% level. Third, again like other countries, the majority of online doctor reviews were positive on the Good Doctor website. And finally, this study shows that the number of doctors may reach a stable level on the Good Doctor website and the number of reviews has been increasing.

### Limitations

This research has limitations. First, all the data used for analysis were from one single website, the Good Doctor, although this website is the largest and the first online doctor reviewing website in China. The website’s design change, database change, and strategy change may affect consumers’ decisions to post a review or not, or to post a positive or negative review. Second, the online doctor reviews were anonymous and there was no way to verify the truthfulness, hence, some of the reviews could have possibly been manipulated with some intention. However, the Good Doctor website does have a policy to check the reliability of reviews by asking the reviewers to leave a phone number on the website, which is not available to the public but only to the webmaster, in order to do random callbacks to verify the truthfulness of the reviews. Third, many doctors received a very limited number of reviews, on average 6 reviews per rated doctor, and those small numbers of reviews may not reflect the reality, or may only partially reflect the reality, of the doctors’ patient populations. Fourth, although China has the largest Internet population in the world, Internet accessibility is still low compared to developed countries around the world—45.8% of the Chinese population has Internet accessibility versus 84% in Germany, 84.2% in the United States, and 89.8% in the United Kingdom [19]. Also, Internet accessibility within China is not equally distributed. On average, about 71% of Internet users are city residents and about 29% are from the countryside, in contrast to 53% of the population being city residents and 47% living in the countryside [32]. Therefore, the digital divide may be preventing many consumers in the countryside from reviewing their doctors online in China.

## Abbreviations

API: application programming interface
G-OB-P: gynecology-obstetrics-pedicatrics
ID: infectious disease
IM: internal medicine
OB: obstetrics
OP: orthopedics
OR: odds ratio
TCM: traditional Chinese medicine
